# Modification of Serine 1040 of SIBRI1 Increases Fruit Yield by Enhancing Tolerance to Heat Stress in Tomato

**DOI:** 10.3390/ijms21207681

**Published:** 2020-10-16

**Authors:** Shufen Wang, Tixu Hu, Aijuan Tian, Bote Luo, Chenxi Du, Siwei Zhang, Shuhua Huang, Fei Zhang, Xiaofeng Wang

**Affiliations:** 1 State Key Laboratory of Crop Stress Biology in Arid Areas, College of Horticulture, Northwest A&F University, Yangling 712100, China; shufenwang@nwafu.edu.cn (S.W.); htx0729@nwsuaf.edu.cn (T.H.); tianaijuan@nwafu.edu.cn (A.T.); robert2018@nwafu.edu.cn (B.L.); cxdu@nwafu.edu.cn (C.D.); Siweizhang@nwafu.edu.cn (S.Z.); shhuang@nwsuaf.edu.cn (S.H.); feizhang@nwafu.edu.cn (F.Z.); 2 Shaanxi Engineering Research Center for Vegetables, Yangling 712100, China

**Keywords:** tomato, SlBRI1, phosphorylation site, heating tolerance, yield

## Abstract

High temperature is a major environmental factor that adversely affects plant growth and production. SlBRI1 is a critical receptor in brassinosteroid signalling, and its phosphorylation sites have differential functions in plant growth and development. However, the roles of the phosphorylation sites of SIBRI1 in stress tolerance are unknown. In this study, we investigated the biological functions of the phosphorylation site serine 1040 (Ser-1040) of SlBRI1 in tomato. Phenotype analysis indicated that transgenic tomato harbouring SlBRI1 dephosphorylated at Ser-1040 showed increased tolerance to heat stress, exhibiting better plant growth and plant yield under high temperature than transgenic lines expressing SlBRI1 or SlBRI1 phosphorylated at Ser-1040. Biochemical and physiological analyses further showed that antioxidant activity, cell membrane integrity, osmo-protectant accumulation, photosynthesis and transcript levels of heat stress defence genes were all elevated in tomato plants harbouring SlBRI1 dephosphorylated at Ser-1040, and the autophosphorylation level of SlBRI1 was inhibited when SlBRI1 dephosphorylated at Ser-1040. Taken together, our results demonstrate that the phosphorylation site Ser-1040 of SlBRI1 affects heat tolerance, leading to improved plant growth and yield under high-temperature conditions. Our results also indicate the promise of phosphorylation site modification as an approach for protecting crop yields from high-temperature stress.

## 1. Introduction

Heat stress is a major abiotic stress that threatens crop production by affecting plant growth processes such as seed germination, root growth, hypocotyl elongation, and fertilization [1]. Physiological and physiochemical analyses have further indicated that heat stress affects photosynthesis and induces excessive reactive oxygen species (ROS) accumulation, which subsequently leads to membrane lipid peroxidation and increased membrane permeability of plants [2]. To avoid heat-induced damage, plants upregulate a series of processes involved in osmotic adjustment, ROS removal, photosynthetic reactions, and saturation of membrane-associated lipids. The genes encoding superoxide dismutase (SOD), peroxidase (POD), and catalase (CAT) participate in ROS scavenging. The major role of heat shock proteins (HSPs) is to act as molecular chaperones regulating protein folding, accumulation, location, and degradation to protect cells against damage due to high-temperature stress. Heat shock factors (HSFs) specifically bind to the heat shock element (HSE) of high-temperature-regulated genes and interact with HSPs to regulate the transcription of genes under high-temperature stress, while the trans-acting *WRKY* factors are overexpressed to help plants respond to high-temperature stress [3,4,5,6,7].

Brassinosteroids (BRs) are a group of steroid hormones that play a potential role in crop yield boosting due to their positive roles in plant growth and tolerance to biotic and abiotic stresses [8,9,10]. BRASSINOSTEROID INSENSITIVE1 (BRI1) is the major BR signalling receptor, and intensive research has confirmed the involvement of BRI1-mediated BR signalling in plant growth and stress responses. In BR signal transduction, the BR first binds to BRI1 to promote its sequential transphosphorylation and heterodimerization with its coreceptor BRI1-ASSOCIATED RECEPTOR KINASE1 (BAK1), which results in the activation of the BR signal [11,12,13]. In *Arabidopsis*, over 30 mutant *bri1* alleles were reported with characteristic BR-insensitive phenotypes, including short hypocotyls in the dark, dwarf stature, prolonged vegetative phase, and male sterility of varying strength; of these, only *bri1-301* additionally exhibited temperature sensitivity [14]. *BRI1* orthologues in different crops, such as rice and tomato, were also found to regulate plant growth and stress tolerance; either loss of function or suppression of *BRI1* usually resulted in shorter plant height, twisted leaves, compromised BR signalling, and altered environmental stress tolerance in plants, while overexpression of *BRI1* could enhance BR signalling to promote plant germination, flowering, and yield [15,16,17,18,19,20]. All these results highlight the critical role of BRI1 in plant growth and stress adaption, and further research on its molecular mechanisms is necessary to realize its valuable potential in crop production.

As a receptor kinase, BRI1 activates BR signal transduction through phosphorylation [11]. Our previous study identified the phosphorylation sites of BRI1 and further revealed their different effects on the biological functions of BRI1 [13,21,22]. Phosphorylation sites in the kinase domain of BRI1 exhibited the strongest functions in BR signalling and plant growth, preventing phosphorylation, which caused severe plant growth inhibition. Phosphorylation sites in the juxtamembrane and C-terminal domains influenced BR signalling to varying degrees, indicating their diverse functions in plant development. For example, dephosphorylation of Ser-1168 and Ser-1172 in the C-terminal domain of BRI1 resulted in slight leaf growth inhibition and greatly reduced seed yields, while dephosphorylation of Tyr-831 in the juxtamembrane domain influenced flowering time and leaf growth [22,23]. In tomato, phosphorylation sites of SlBRI1 were also found to be critical for SlBRI1-mediated regulation of BR signal transduction and plant biological development. Dephosphorylation of Thr-1054 in SlBRI1 severely attenuated BR signalling and disturbed plant growth in tomato [24]. Our previous study further found that the tomato SlBRI1 phosphorylation site Thr-1050 could affect tomato yield by regulating BR signalling [25]. These results suggest the potential value of phosphorylation sites of BRI1 in crop agronomic trait improvement, given their precise modification of the biological function of BRI1. However, functional analyses of the phosphorylation sites of SlBRI1 are limited and focus on plant growth, while the functions of these sites in stress tolerance are still unknown.

In this work, we investigated the biological functions of the phosphorylation site serine 1040 (Ser-1040) of SlBRI1 in tomato. The weak SlBRI1 mutant *cu3^-abs1^* (the result of a His-1012-Tyr (H1012Y) missense mutation in SlBRI1), as well as *cu3^-abs1^* transformed with SlBRI1 constitutively phosphorylated at Ser-1040, SlBRI1 dephosphorylated at Ser-1040, or wild-type SlBRI1, was used for phenotype comparison. Our results showed that transgenic plants harbouring dephosphorylated Ser-1040 exhibited similar growth phenotypes under normal temperature conditions but better growth and more efficient stress responses than plants harbouring phosphorylated Ser-1040 or SlBRI1 under high-temperature conditions. Furthermore, compared with wild-type SlBRI1 and SlBRI1 phosphorylated at Ser-1040, dephosphorylation of Ser-1040 resulted in a lower autophosphorylation level of SlBRI1 in vitro but a similar BR signal strength in plants. These results suggest that Ser-1040 can modulate BRI1 autophosphorylation to promote plant heat tolerance. Our results provide a theoretical basis for revealing the molecular modulation mechanism of SlBRI1 in stress adaption, as well as coordinating tomato yield with environmental changes through fine-tuning of the phosphorylation site of SlBRI1.

## 2. Results

### 2.1. SlBRI1 Ser-1040 Influences Autophosphorylation of SlBRI1

**Figure 1:**
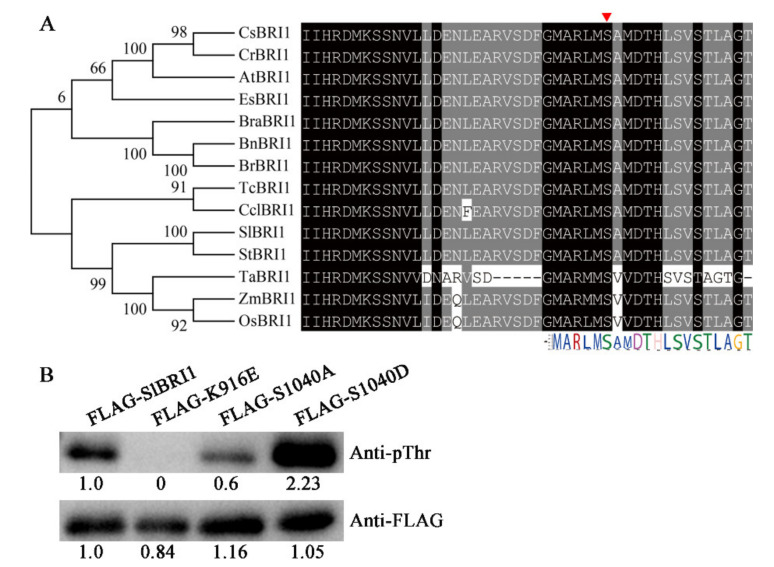
SlBRI1 Ser-1040 influences autophosphorylation of SlBRI1. (**A**) Alignment of the partial kinase domain sequences of BRI1 homologues. Conserved and similar residues were highlighted with black and gray grounds, respectively. The set of Ser-1040 among BRI1 homologues was marked by the red arrow. Each symbol with different colors at the bottom indicated the conservation of residue at each site. SlBRI1 (*Solanum lycopersicum*, NP_001296180.1), CsBRI1 (*Camelina sativa*, XP_010431911.1), CrBRI1 (*Camelina sativa*, XP_010431911.1), AtBRI1 (*Arabidopsis thaliana*, NP_195650.1), EsBRI1 (*Eutrema salsugineum*, XP_006411743.1), BraBRI1 (*Brassica oleracea* var. oleracea, XP_013597742.1), BnBRI1 (*Brassica napus*, NP_001303105.1), BrBRI1 (*Brassica rapa XP_009101880.2*), TcBRI1 (*Theobroma cacao*, XP_017985424.1), CclBRI1 (*Citrus clementina*, XP_006427932.1), StBRI1 (*Solanum tuberosum*, XP_006357355.1), TaBRI1 (*Triticum aestivum*, DQ_655711.1), ZmBRI1 (*Zea mays*, XP_008656807.1) and OsBRI1 (*Oryza sativa*, NP_001044077.1). (**B**) Autophosphorylation level of SlBRI1 in vitro. Autophosphorylation analysis of recombinant FLAG-SlBRI1, FLAG-K916E, FLAG-S1040A, and FLAG-S1040D proteins was detected by anti-pThr antibodies, and anti-FLAG antibodies were used to show the loading levels for western blotting. Intensities of bands were presented as relative values compared with the FLAG-SlBRI1.

SlBRI1 is a receptor kinase that transmits BR signals through phosphorylation [24]. To determine the function of SlBRI1 Ser-1040 in tomato, we first explored the conservation of Ser-1040. Protein sequence alignment of BRI1 homologues revealed that Ser-1040 was a highly conserved phosphorylation site among the distinct species. This high conservation indicated that Ser-1040 had a crucial role in the biological function of SlBRI1 (Figure 1A).

Autophosphorylation of SlBRI1 is usually important for its functions in plants, and most of the phosphorylation sites of SlBRI1 positively regulate the autophosphorylation. To determine whether the phosphorylation site Ser-1040 could influence the autophosphorylation of SlBRI1, we compared the autophosphorylation levels of SlBRI1, a kinase-inactive form of SlBRI1 (K916E, in which Lys-916 was replaced with glutamic acid), S1040A (in which Ser-1040 of SlBRI1 was replaced with alanine), and S1040D (in which Ser-1040 was replaced with aspartic acid) in vitro. The results suggested a positive role for Ser-1040 phosphorylation in SlBRI1 autophosphorylation, since the intensity of the phosphorylation band of FLAG-S1040D was strongest, the phosphorylation level of which was 4.1- and 2.1-fold that of FLAG-S1040A and FLAG-SlBRI1, respectively (Figure 1B). Thus, dephosphorylation of Ser-1040 might attenuate kinase active of SlBRI1, and further disturb the biological functions.

### 2.2. SlBRI1 Ser-1040 Slightly Affects BR Signalling in Tomato

**Figure 2:**
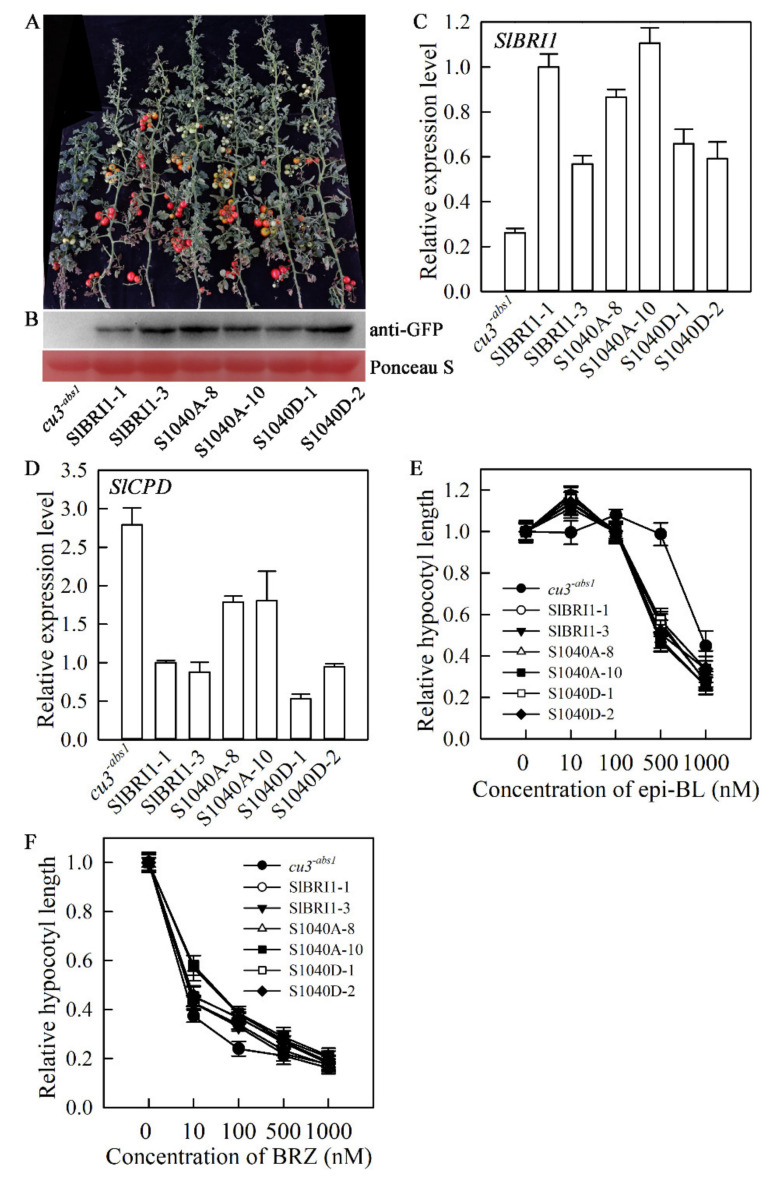
SlBRI1 Ser-1040 slightly affects BR signalling in tomato. (**A**) Phenotypes of plants at the maturation stage. The plants shown from left to right are as follows: *cu3^-abs1^*, SlBRI1-1, SlBRI1-3, S1040A-8, S1040A-10, S1040D-1, and S1040D-2. (**B**) Western blot analysis of transgenic protein expression using anti-green fluorescent protein (GFP) antibodies. Ponceau S (Solarbio, P8330, Beijing, China) staining shows loading. (**C**) and (**D**) Relative transcript levels of SlBRI1 (**C**) and the BR signalling marker gene *SlCPD* (**D**) in tomato. (**E**) and (**F**) Dose-response curves of relative hypocotyl lengths of tomato seedlings grown in the dark for 10 days on the surface of media with increasing concentrations of epi-BL (**E**) and BRZ (**F**). The data for (**E**) and (**F**) are the means ± SDs of 15 independent biological samples.

To investigate the biological function of SlBRI1 Ser-1040 in tomato, transgenic plants in the *cu3^-abs1^* background expressing SlBRI1, S1040A, or S1040D were generated for phenotype evaluation (Figure 2A). The transgenic lines P*_SlBRI1_*::SlBRI1-GFP-1 and P*_SlBRI1_*::SlBRI1-GFP-3 (SlBRI1-1 and SlBRI1-3 for short) were selected as the positive controls, while the weak *SlBRI1* mutant *cu3^-abs1^* was considered the negative control. Both protein level and transcription level analyses suggested that all transgenic lines used in this study had a higher level of SlBRI1 than *cu3^-abs1^* plants (Figure 2B,C).

Previous studies have suggested that the BR biosynthesis gene *SlCPD* could be suppressed by BR signalling feedback; thus, the expression of this gene could be considered to be a marker of BR signal strength [26]. Consistent with this feedback regulation, the transcript levels of *SlCPD* in P*_SlBRI1_*::SlBRI1-GFP and P*_SlBRI1_*::S1040D-GFP (S1040D-1 and S1040D-2 for short) plants were similar and lower than those in *cu3^-abs1^* plants, while in P*_SlBRI1_*::S1040A-GFP (S1040A-8 and S1040A-10 for short) lines, the levels were between those in P*_SlBRI1_*::SlBRI1-GFP and *cu3^-abs1^* plants, which suggested that both wild-type SlBRI1 and SlBRI1 with phosphorylated Ser-1040 could rescue the BR signalling defect in *cu3^-abs1^* plants, and the BR signal strength in P*_SlBRI1_*::S1040A-GFP lines was slightly weaker than that in P*_SlBRI1_*::SlBRI1-GFP and P*_SlBRI1_*::S1040D-GFP lines (Figure 2D).

The BR sensitivity of the seedlings was also analysed to further quantitatively evaluate the BR signal strength among *cu3^-abs1^*, P*_SlBRI1_*::SlBRI1-GFP, P*_SlBRI1_*::S1040A-GFP, and P*_SlBRI1_*::S1040D-GFP plants. The hypocotyl lengths of the transgenic lines and *cu3^-abs1^* were measured, and their relative hypocotyl lengths under five increasing concentrations of exogenous 24-epibrassinolide (epi-BL) or BR inhibitor brassinazole (BRZ) were compared. As shown in Figure 2E and 2F, *cu3^-abs1^* was insensitive to epi-BL and sensitive to BRZ, since the hypocotyl length this plant was nearly unchanged when the concentration of epi-BL was lower than 500 nM, while a 62.7% decrease was observed under 10 nM BRZ. However, the change rates of the hypocotyl length among the transgenic plants were similar regardless of treatment with epi-BL or BRZ. This result demonstrated that Ser-1040A could rescue BR signal transduction in tomato.

### 2.3. Dephosphorylation of Ser-1040 Improves Tomato Yield under Heat Stress

**Figure 3:**
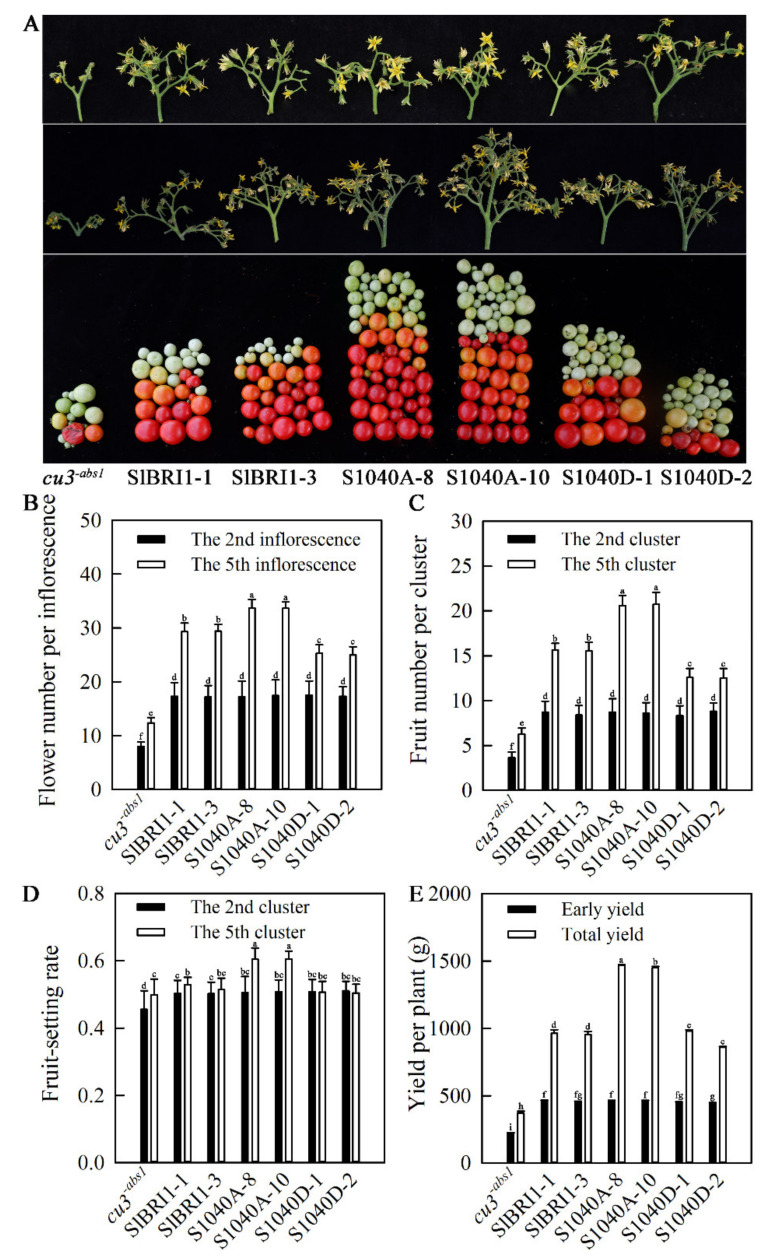
Dephosphorylation of Ser-1040 improves tomato yield under heat stress. (**A**) Phenotypes of the second inflorescence (top), fifth inflorescence (middle) and total yield per plant (bottom). (**B**) Flower numbers of the second and fifth inflorescences. (**C**) Fruit number and (**D**) fruit setting rate of the second and fifth clusters. (**E**) Early yield and total yield per plant. The different letters indicate significant differences at the 0.05 level. The data for (**B**) and (**E**) are the means ± SDs of 10 independent biological samples.

To assess the effect of Ser-1040 on tomato yield under normal and heat stress conditions, plant height and stem diameter at the mature stage; flower number, stamen length, pistil length, and flower phenotype of the second and fifth inflorescences; fruit number and fruit setting rate of the second and fifth clusters; single-fruit weight; and early yield and total yield per plant were compared. As shown in Figure 3A and Figure 3B, the flower numbers of the second inflorescences that grew at normal temperature were similar; however, the fifth inflorescences that grew in the late-spring stage with high temperature showed a significant difference among the transgenic lines. The flower numbers of the fifth inflorescences of P*_SlBRI1_*::S1040A-GFP lines were the largest, approximately 12.9% and 25.3% larger than those of P*_SlBRI1_*::SlBRI1-GFP and P*_SlBRI1_*::S1040D-GFP lines, respectively. Fruit numbers per second cluster were similar among the transgenic lines, whereas the fruit number per fifth cluster of P*_SlBRI1_*::S1040A-GFP lines were 24.5% and 39.2% higher than those of P*_SlBRI1_*::SlBRI1-GFP and P*_SlBRI1_*::S1040D-GFP lines (Figure 3C). In terms of fruit setting rate, the second clusters from all transgenic lines were the same, while the fifth clusters were also constant for P*_SlBRI1_*::SlBRI1-GFP and P*_SlBRI1_*::S1040D-GFP plants; however, P*_SlBRI1_*::S1040A-GFP lines showed higher fruit setting rates of the fifth cluster, the values for which were 13.8% and 16.4% greater than those for P*_SlBRI1_*::SlBRI1-GFP and P*_SlBRI1_*::S1040D-GFP lines (Figure 3D). Furthermore, the early yield per plant for each transgenic line was similar, while P*_SlBRI1_*::S1040D-GFP lines exhibited 1.52-fold and 1.59-fold higher total yields per plant than P*_SlBRI1_*::SlBRI1-GFP and P*_SlBRI1_*::S1040D-GFP lines, respectively (Figure 3A,E). Furthermore, plant height, stem diameter, and single-fruit weight at the mature stage were similar among the transgenic lines (Figures S1A–C). Phenotype analysis of the second and fifth inflorescences showed that there were no significant differences in flower phenotype and stamen length among the transgenic plants under either normal or high-temperature conditions. However, the pistils were nearly the same under normal temperature conditions but showed different extension trends under high temperature, and this extension was most obvious in P*_SlBRI1_*::S1040D-GFP lines compared with other transgenic lines (Figures S1D–F). In conclusion, Ser-1040 appears to play an important role in tomato fruiting under high temperature.

### 2.4. Dephosphorylation of Ser-1040 Promotes Germination and Seedling Growth under Heat Stress

**Figure 4:**
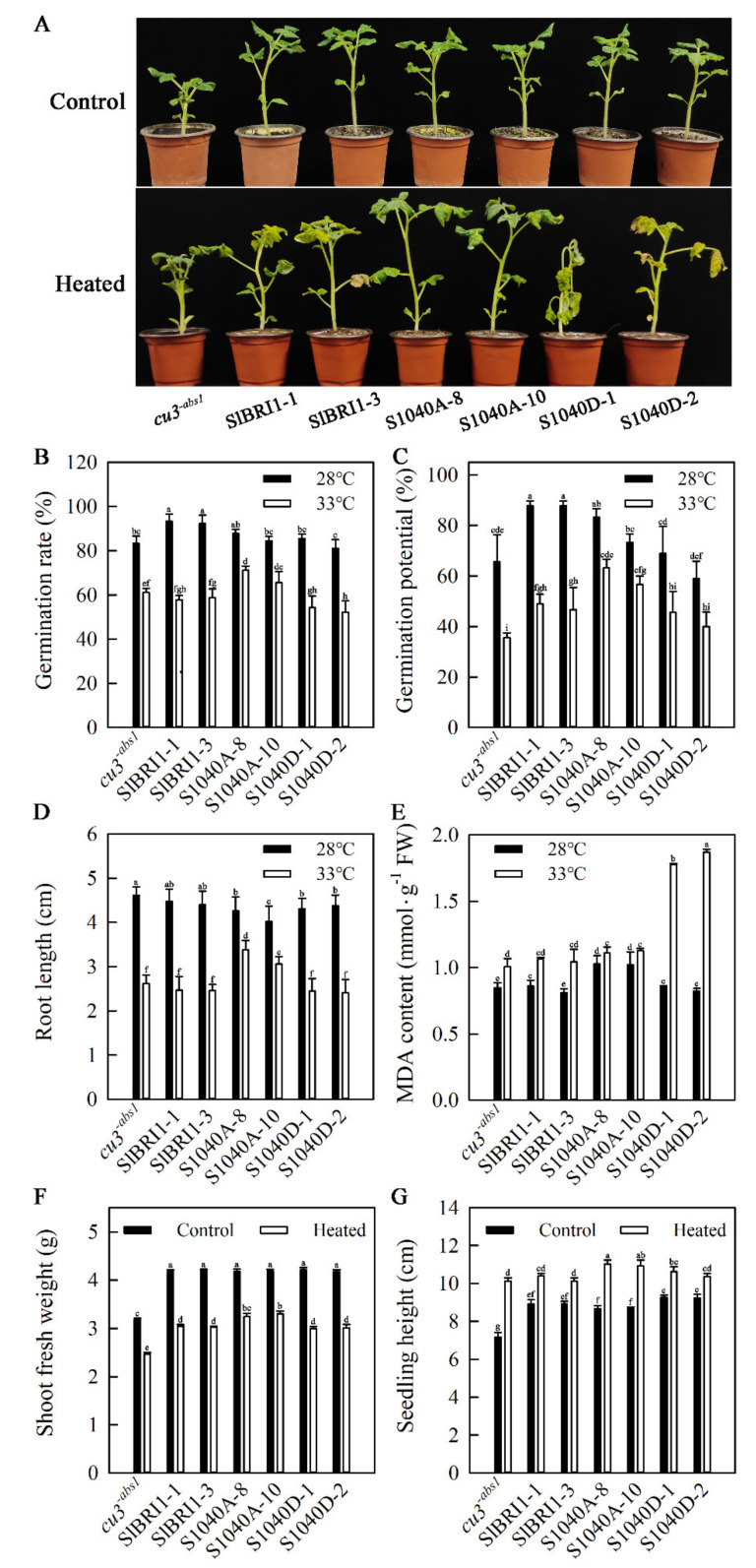
Dephosphorylation of Ser-1040 promotes germination and seedling growth under heat stress. (**A**) Phenotypes of seedlings treated with or without heat stress for 9 days. (**B**) Germination rate of plants on the fourteenth day after seeding at 33 °C or 28 °C. (**C**) Germination potential of plants on the fourth day after seeding at 33 °C or 28 °C. (**D**) Root length and (**E**) MDA content of plants on the fourteenth day after seeding at 33 °C or 28 °C. (**F**) Shoot fresh weights and (**G**) seedling heights of plants at the four-leaf stage treated with or without heat stress (38 °C/28 °C, day/night) for 12 days. The data for (**B**) and (**E**) are the means ± SDs of three replicates, and each replicate had 30 plants. The data for (**F**) and (**G**) are the means ± SDs of three independent biological samples. The different letters indicate significant differences at the 0.05 level.

To determine how Ser-1040 phosphorylation extensively affects seed germination under heat stress, the seed germination phenotypes of transgenic lines and *cu3^-abs1^* were analysed. The difference in germination rates among transgenic lines was not obvious at 28 °C; however, the germination rates of P*_SlBRI1_*::S1040A-GFP lines were 1.17-fold and 1.28-fold higher than those of P*_SlBRI1_*::SlBRI1-GFP and P*_SlBRI1_*::S1040D-GFP lines when the temperature increased to 33 °C (Figure 4B). The germination potential of P*_SlBRI1_*::SlBRI1-GFP lines decreased most dramatically, by approximately half, while the decreases in germination potentials of P*_SlBRI1_*::S1040D-GFP and P*_SlBRI1_*::S1040A-GFP lines were 33.0% and 23.4%, respectively, when the temperature increased from 28 °C to 33 °C (Figure 4C). The sensitivity of root growth to heat stress was also analysed. P*_SlBRI1_*::S1040A-GFP plants were insensitive to heat stress, and their root lengths decreased by 22.3% at 33 °C, while those of P*_SlBRI1_*::SlBRI1-GFP and P*_SlBRI1_*::S1040D-GFP plants decreased by 44.5% and 44.1%, respectively (Figure 4D). At 28 °C, the malondialdehyde (MDA) content in P*_SlBRI1_*::S1040A-GFP leaves was higher than that in other lines. When the germination temperature was 33 °C, the MDA content in all plants increased to different degrees, and the MDA content in P*_SlBRI1_*::S1040A-GFP plants increased slightly and was similar to that in P*_SlBRI1_*::SlBRI1-GFP plants, while in P*_SlBRI1_*::S1040D-GFP plants, the MDA content increased rapidly by more than 1.6-fold compared with that in other lines (Figure 4E).

Tomato seedlings of transgenic lines and *cu3^-abs1^* at the four-leaf stage were exposed to 38 °C/28 °C or 25 °C/25 °C for 9 days, and seedling phenotypes, shoot fresh weights, shoot dry weights, seedling heights and seedling stem diameters were subsequently analysed to determine how Ser-1040 phosphorylation affects tomato seedling growth under heat stress. As shown in Figure 4A, the values for all the transgenic plants were the same and were higher than those for *cu3^-abs1^* plants under normal conditions. When the seedlings were exposed to 38 °C/28 °C (day/night) for 9 days, P*_SlBRI1_*::S1040D-GFP and P*_SlBRI1_*::SlBRI1-GFP plants showed more severe wilting phenotypes than P*_SlBRI1_*::S1040A-GFP and *cu3^-abs1^* plants. Shoot fresh weights among the transgenic lines were nearly the same and higher than those of *cu3^-abs1^* before heat stress. Following heat stress, the decreases in shoot fresh weights of P*_SlBRI1_*::S1040A-GFP plants were 21.0% and 23.4% lower than those of P*_SlBRI1_*::SlBRI1-GFP and P*_SlBRI1_*::S1040D-GFP plants (Figure 4F). The shoot dry weights showed similar changes when the temperature increased (Figure S2A). The seedling heights of P*_SlBRI1_*::SlBRI1-GFP and P*_SlBRI1_*::S1040D-GFP plants were similar and slightly higher than those of P*_SlBRI1_*::S1040A-GFP plants when the temperature was 25 °C. However, P*_SlBRI1_*::S1040A-GFP lines showed the second-fastest growth rates after *cu3^-abs1^* at high temperature, approximately 38.5% and 43.6% faster than those of P*_SlBRI1_*::SlBRI1-GFP and P*_SlBRI1_*::S1040D-GFP lines (Figure 4G). In addition, P*_SlBRI1_*::S1040A-GFP lines also exhibited the lowest decline rates for the seedling stem diameter compared with P*_SlBRI1_*::SlBRI1-GFP and P*_SlBRI1_*::S1040D-GFP plants (Figure S2B).

### 2.5. Dephosphorylation of Ser-1040 Promotes Heat Stress Tolerance of Seedlings

**Figure 5:**
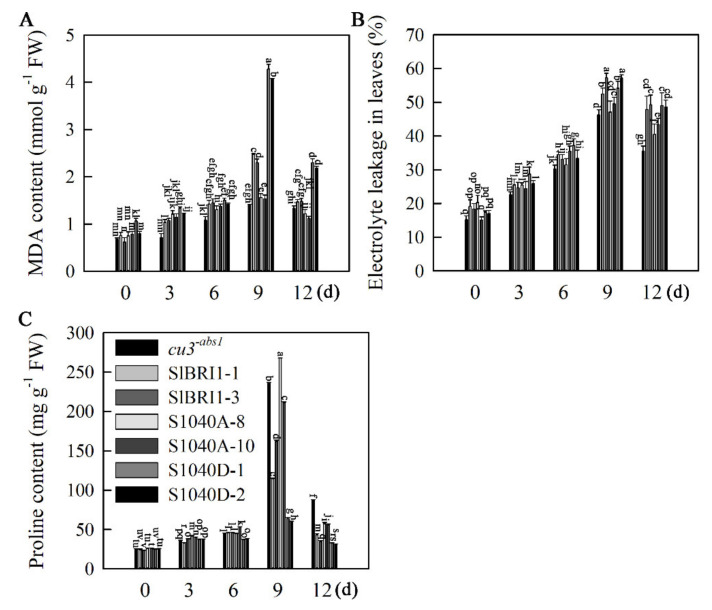
Dephosphorylation of Ser-1040 promotes the heat stress tolerance of seedlings. (**A**–**C**) Time course of changes in MDA content (**A**), ion leakage (**B**), and proline content (**C**) in tomato seedlings in response to heat stress. Tomato seedlings at the four-leaf stage were placed into a growth chamber set at 38 °C/28 °C (day/night) for 9 days and then transferred to 25 °C for 3 days. The data are the means ± SDs of at least three independent biological samples. The different letters indicate significant differences at the 0.05 level.

To determine whether Ser-1040 phosphorylation affects heat tolerance in tomato, the free proline accumulation, electrolyte leakage and MDA content, which are indicators of cell membrane damage caused by heat stress, were measured. As shown in Figure 5A, the MDA content increased in all of the seedlings after heat stress and was highest in P*_SlBRI1_*::S1040D-GFP lines, approximately 1.8, 2.7 and 2.9 times that in P*_SlBRI1_*::SlBRI1-GFP, P*_SlBRI1_*::S1040A-GFP, and *cu3^-abs1^* plants, respectively, on the ninth day of heat stress. Similarly, the levels of electrolyte leakage in P*_SlBRI1_*::S1040D-GFP and P*_SlBRI1_*::SlBRI1-GFP leaves were significantly higher than those in P*_SlBRI1_*::S1040A-GFP and *cu3^-abs1^* plants after heat stress (Figure 5B). In contrast, the proline content was lowest in P*_SlBRI1_*::S1040D-GFP lines, approximately 44.6%, 25.8% and 26.2% that in P*_SlBRI1_*::SlBRI1-GFP, P*_SlBRI1_*::S1040A-GFP, and *cu3^-abs^* plants, under treatment with high temperature for nine days (Figure 5C).

### 2.6. Dephosphorylation of Ser-1040 Promotes ROS Detoxification of Seedlings under Heat Stress

**Figure 6:**
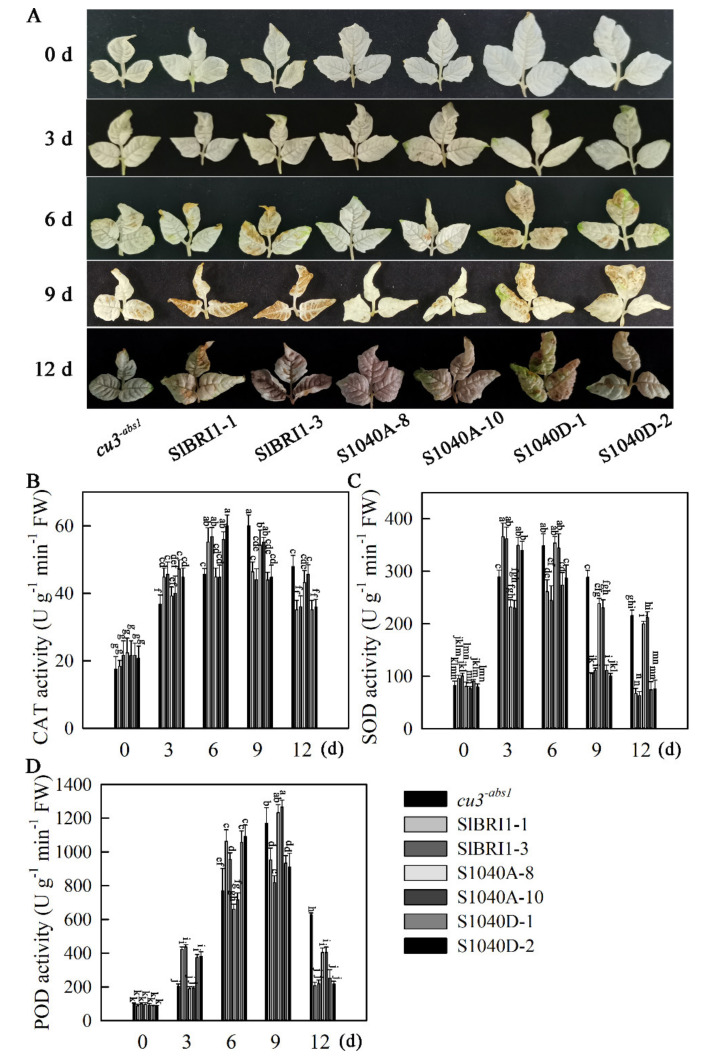
Dephosphorylation of Ser-1040 promotes ROS detoxification in seedlings under heat stress. (**A**) DAB staining for hydrogen peroxide (H_2_O_2_) in leaves from tomato seedlings during heat stress treatment. (**B**–**D**) Time course of changes in CAT (**B**), SOD (**C**), and POD (**D**) activities in tomato seedlings in response to heat stress. Tomato seedlings at the four-leaf stage were placed into a growth chamber set at 38 °C/28 °C (day/night) for 9 days and then transferred to 25 °C for 3 days. The data for (**B**) to (**D**) are the means ± SDs of at least three replicates, and each replicate had 10 plants. The different letters indicate significant differences at the 0.05 level.

The activity of antioxidants is important in the protection of plants from ROS-induced damage under exposure to heat stress [27]. To investigate whether Ser-1040 phosphorylation affects heat stress tolerance though altered redox status, H_2_O_2_ accumulation, and antioxidant enzyme activities, the expression levels of antioxidant-related genes in *cu3^-abs1^* and transgenic seedlings under normal and high temperature were determined. As shown in Figure 6A, all of the plants accumulated nearly equal amounts of basal H_2_O_2_ when grown under normal conditions. Under high temperature, the H_2_O_2_ content in these plants decreased in the following order: P*_SlBRI1_*::S1040D-GFP, P*_SlBRI1_*::SlBRI1-GFP, P*_SlBRI1_*::S1040A-GFP and *cu3^-abs1^*. In the antioxidant enzyme activity analysis, CAT activity in all plants showed similar peak values during heat stress; however, CAT activity peaked more quickly in P*_SlBRI1_*::SlBRI1-GFP and P*_SlBRI1_*::S1040D-GFP lines, showing rapid spikes on the sixth day of heat stress, three days earlier than those observed in P*_SlBRI1_*::S1040A-GFP and *cu3^-abs1^* plants (Figure 6B). SOD activity in P*_SlBRI1_*::SlBRI1-GFP and P*_SlBRI1_*::S1040D-GFP transgenic lines peaked on the third day of heat stress and then declined rapidly. While SOD activity in P*_SlBRI1_*::S1040A-GFP and *cu3^-abs1^* plants peaked on the sixth day of heat stress and the decline was relatively slow, SOD activity in P*_SlBRI1_*::S1040A-GFP plants was more than 2.5-fold that in P*_SlBRI1_*::SlBRI1-GFP and P*_SlBRI1_*::S1040D-GFP lines after heat stress (Figure 6C). POD activity in P*_SlBRI1_*::SlBRI1-GFP and P*_SlBRI1_*::S1040D-GFP plants was higher until the sixth day of heat stress but was then surpassed by that in P*_SlBRI1_*::S1040A-GFP and *cu3^-abs1^* plants. The peak value of POD activity in P*_SlBRI1_*::S1040A-GFP plants was at least 10% higher than those in P*_SlBRI1_*::SlBRI1-GFP and P*_SlBRI1_*::S1040D-GFP lines (Figure 6D).

### 2.7. Dephosphorylation of Ser-1040 Promotes Photosynthesis in Seedlings under Heat Stress

**Figure 7:**
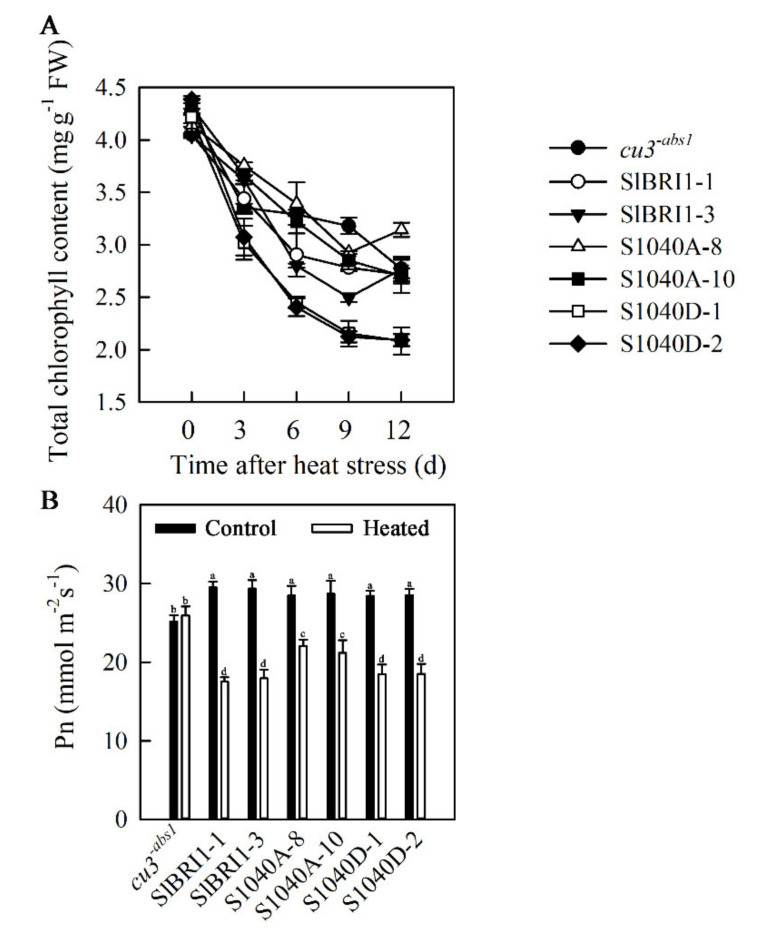
Dephosphorylation of Ser-1040 promotes photosynthesis in seedlings under heat stress. (**A**) Time course of changes in total chlorophyll content in tomato seedlings in response to heat stress. (**B**) CO_2_ assimilation rate in tomato at the flowering stages of the second inflorescence (control) and fourth inflorescence (heated). The data are the means ± SDs of at least 3 replicates, and each replicate had 10 plants. The different letters indicate significant differences at the 0.05 level.

Photosynthesis in plants is sensitive to high temperature and is usually inhibited under heat stress [28]. To determine whether Ser-1040 phosphorylation affects tomato photosynthesis under heat stress, the total chlorophyll content and CO_2_ assimilation rate (Pn) of *cu3^-abs1^* and transgenic plants under normal and high temperatures were determined. The total chlorophyll content in all the plants decreased under heat stress; however, P*_SlBRI1_*::S1040D-GFP exhibited the greatest decline at 51.4%. P*_SlBRI1_*::S1040A-GFP showed the slowest decline, with the value decreasing by 30.8% on the twelfth day of heat stress (Figure 7A). The CO_2_ assimilation rates of all the transgenic plants were the same at the flowering stage of the second inflorescence and decreased at the flowering stage of the fifth inflorescence. The decline rates of P*_SlBRI1_*::S1040A-GFP lines were the lowest and only approximately 61.3% and 69.3% those of P*_SlBRI1_*::SlBRI1-GFP and P*_SlBRI1_*::S1040D-GFP lines, respectively (Figure 7B).

### 2.8. Dephosphorylation of Ser-1040 Enhances the Expression of Tomato Defence-Related Genes under Heat Stress

**Figure 8:**
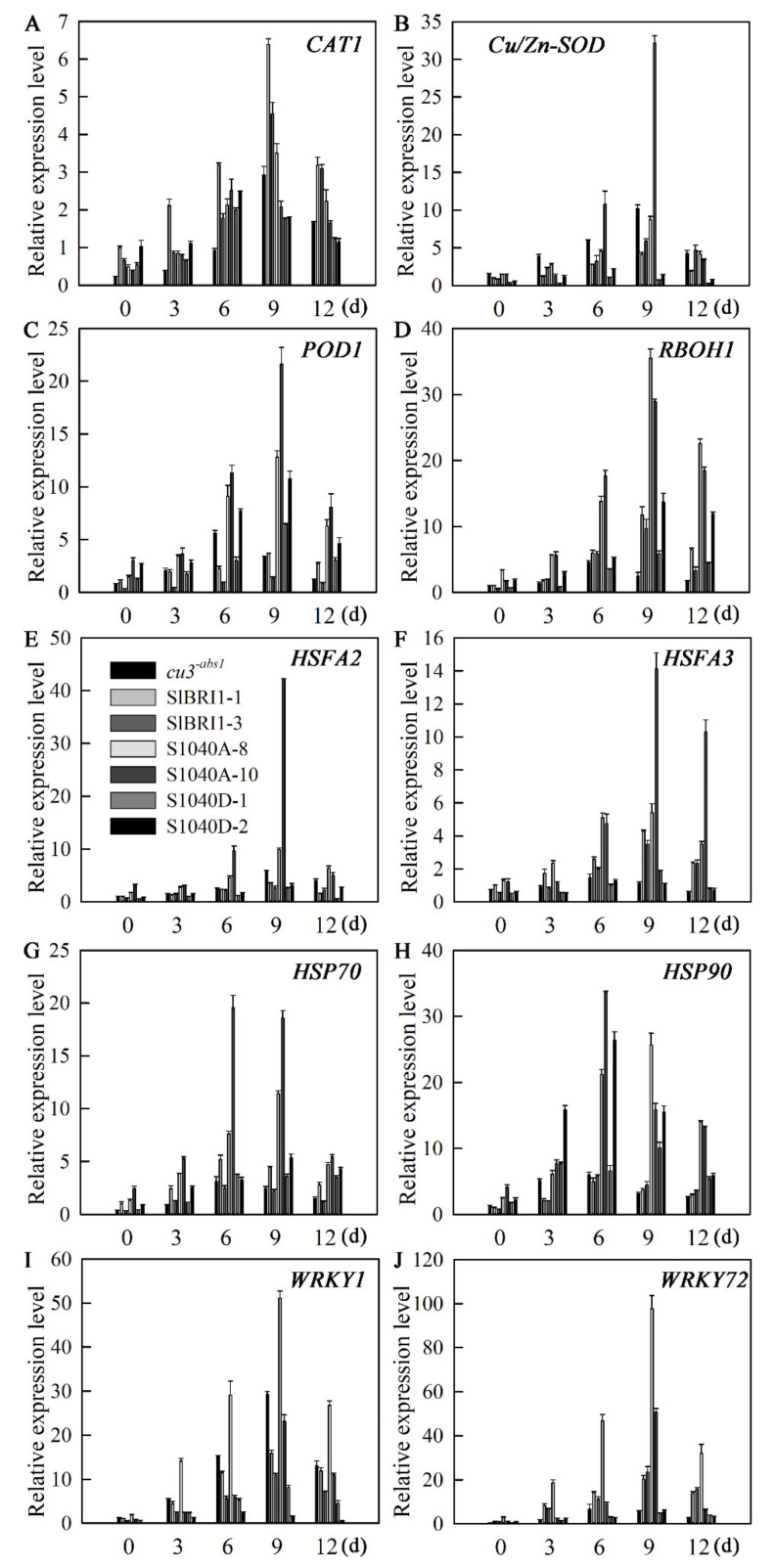
Dephosphorylation of Ser-1040 enhances the expression of tomato defence-related genes under heat stress. (**A**–**J**) Transcription levels of *CAT1* (**A**), *Cu/Zn-SOD* (**B**), *POD1* (**C**), *RBOH1* (**D**), *HSFA2* (**E**), *HSFA3* (**F**), *HSP70* (**G**), *HSP90* (**H**), *WRKY1* (**I**), and *WRKY72* (**J**) in tomato seedlings in response to heat stress. Tomato seedlings at the four-leaf stage were placed into a growth chamber set at 38 °C/28 °C (day/night) for 9 days and then transferred to 25 °C for 3 days. The data are the means ± SDs of three biological replicates and technical replications.

To reveal the molecular mechanisms underlying the role of Ser-1040 phosphorylation in tomato heat tolerance, the transcription levels of defence-related genes, such as *CAT1*, *Cu/Zn-SOD*, *POD1*, *HSPs*, *HSFs* and *WRKYs,* were analysed at 0, 3, 6, 9, and 12 days under heat stress. The differences in the transcripts of antioxidant-related genes, such as *CAT1*, *Cu/Zn-SOD*, and *POD1,* were nonsignificant when the transgenic plants were grown under normal conditions, and heat stress markedly induced the expression of all the analysed antioxidant-related genes. With prolonged heating, the increase in expression of *Cu/Zn-SOD*, *POD1*, and *RBOH* in P*_SlBRI1_*::S1040A-GFP plants was significantly higher than that in P*_SlBRI1_*::SlBRI1-GFP and P*_SlBRI1_*::S1040D-GFP plants (Figure 8A–D). The transcription levels of *HSFA2* in P*_SlBRI1_*::SlBRI1-GFP and P*_SlBRI1_*::S1040D-GFP transgenic lines were unchanged under heat stress; however, in P*_SlBRI1_*::S1040A-GFP transgenic lines, the transcription increased rapidly and peaked on the ninth day of heat stress (Figure 8E). The expression of *HSFA3* in P*_SlBRI1_*::S1040D-GFP lines was unchanged under heat stress; however, it increased in P*_SlBRI1_*::SlBRI1-GFP and P*_SlBRI1_*::S1040A-GFP lines, and P*_SlBRI1_*::S1040A-GFP lines showed the greatest increase when exposed to high-temperature conditions (Figure 8F). The expression of *HSP70* and *HSFA90* in all the plants increased and peaked on the sixth day of heat stress, while P*_SlBRI1_*::S1040A-GFP lines showed the greatest increase compared with P*_SlBRI1_*::SlBRI1-GFP and P*_SlBRI1_*::S1040D-GFP lines (Figure 8G,H). The expression of *WRKY1* and *WRKY72* in all the plants increased and peaked on the ninth day of heat stress, and again, P*_SlBRI1_*::S1040A-GFP lines showed the greatest increase among all the plants, while P*_SlBRI1_*::S1040D-GFP lines showed the lowest increase (Figure 8I,J).

## 3. Discussion

High temperature is one of the most serious problems for agricultural production, and BRs are endogenous plant hormones that are involved in the processes of plant growth and environmental stress adaption [8]. As a BR receptor, the functions of BRI1 in plant growth are conserved and well established; however, its functional mechanism for adjusting plant growth and development according to heat stress remains largely unknown. Previous studies in *Arabidopsis* found that increased temperature specifically impacts BRI1 expression to downregulate BR signalling and mediate root elongation [28]. The *Arabidopsis bri1-301* mutant exhibited severe growth defects when the temperature increased from 22 °C to 29 °C [14]. In tomato, the SlBRI1 mutant *cu3^-abs1^* seedlings showed high basal tolerance to heat stress [29]. In this study, we first found that the phosphorylation site of SlBRI1 could influence its functions on stress tolerance, and dephosphorylation of Ser-1040 significantly increased the capability of tomato to maintain growth parameters and maintained a stable yield under heat stress. Compared with the transgenic line with wild-type SlBRI1 and the line with SlBRI1 phosphorylated at Ser-1040, plants harbouring dephosphorylated Ser-1040 exhibited a higher yield, more stable photosynthesis, a more active ROS-scavenging system, and higher expression levels of stress defence genes. Previous studies have suggested that Ser-1040 is located in the activation loop of SlBRI1, and dephosphorylation of Ser-1040 almost completely abolishes the kinase activity of SlBRI1 in vitro [24]. Consistent with this, our results showed that Ser-1040 acts as a positive regulator of SlBRI1 kinase activity, since dephosphorylated and phosphorylated Ser-1040 showed decreased and increased phosphorylation levels compared with that of wild-type SlBRI1 in vitro (Figure 1). However, the BR signal strengths in P*_SlBRI1_*::S1040A-GFP, P*_SlBRI1_*::S1040D-GFP and P*_SlBRI1_*::SlBRI1-GFP were similar. This raises the question of whether phosphorylation sites of BRI1 influence its functions only through classic BR signal transduction. Previous studies conducted in *Arabidopsis* reported that dephosphorylation of Thr-872 of BRI1 resulted in a dramatic increase in BRI1 phosphorylation levels in vitro; however, transgenic plants with dephosphorylated Thr-872 did not show the strong performance exhibited by plants expressing wild-type BRI1. In addition, phosphorylation sites in the C-terminal domain of BRI1 did not influence BR signalling but could regulate the reproductive development of plants [22]. Thus, phosphorylation sites might influence BRI1 function not only by influencing BR signalling but also by influencing other BR-induced pathways through changing posttranslational modifications, such as protein interactions, folding, localization, and degradation. Furthermore, the molecular mechanism of the tomato BR signal transduction pathway remains unclear, and the relevant indicators are still limited. All these results demonstrated that Ser-1040 regulated tomato heat tolerance mainly by affecting the kinase activity of SlBRI1, and this pattern was distinct from the classic BR signal pathway.

High temperature triggers lipid peroxidation, which could be marked by ROS accumulation. During this process, both respiratory burst oxidase homologue (RBOH) and antioxidant enzymes such as CAT, SOD, and POD play pivotal roles in plants, helping them to adapt to environmental stress by ROS production and scavenging, respectively [30,31]. Studies on BR signalling mutants of *Arabidopsis*, tomato and other crops revealed that BRs play a protective role under high temperature, as they promote antioxidant enzyme activities and elevate *RBOH1* transcription [27,32]. In tomato, overexpression of *BZR1* enhanced the transcription of *RBOH1* and subsequently elevated heat tolerance, and seedling of the SlBRI1 mutant *cu3^-abs1^* exhibited induction of thermotolerance, showing increased signs of oxidative stress [29,33]. In this study, P*_SlBRI1_*::S1040A-GFP lines that were tolerant to heat stress showed higher expression levels of *RBOH1* but lower accumulation of H_2_O_2_ than P*_SlBRI1_*::S1040D-GFP and P*_SlBRI1_*::SlBRI1-GFP plants after heat stress (Figure 6 and Figure 8). The reduced accumulation of H_2_O_2_ in P*_SlBRI1_*::S1040A-GFP lines resulted from increases in activities and transcription of ROS-scavenging enzymes, namely, CAT, SOD, and POD, and the increased activities of these ROS-scavenging enzymes were caused by increased expression levels of *Cu/Zn-SOD* and *POD1* (Figure 6 and Figure 8). The cell membrane is the major target of heat injury and is sensitive to lipid peroxidation. P*_SlBRI1_*::S1040A-GFP lines, with the highest activities of the ROS-scavenging system, showed less membrane damage that P*_SlBRI1_*::S1040D-GFP and P*_SlBRI1_*::SlBRI1-GFP plants after heat stress, as the MDA content and electrolyte leakage were lower in P*_SlBRI1_*::S1040A-GFP lines, while the proline content was higher (Figure 5). Numerous studies have demonstrated that chlorophyll, which is the most important pigment for photosynthesis, is inhibited by heat stress [34]. Under high-temperature conditions, P*_SlBRI1_*::S1040A-GFP lines showed higher chlorophyll content and CO_2_ assimilation rates than P*_SlBRI1_*::S1040D-GFP and P*_SlBRI1_*::SlBRI1-GFP plants (Figure 7). These results clearly suggested that dephosphorylation of SlBRI1 Ser-1040 could protect tomato plants from ROS-induced cellular injury and sustain their photosynthetic capacity to enhance their heating tolerance.

High-temperature tolerance in plants has largely been achieved by elevating the expression of *HSP* genes and of trans-acting factors such as HSFs and *WRKYs*. Previous studies demonstrated that HSP70 and HSP90 are abundant in eukaryotic cells and responsible for cell protection under high-temperature conditions [35,36,37]. The *HvBRI1* mutant of barley was characterized by decreased accumulation of *HSP*s under high temperature [38]. In tomato, overexpressing *HSP21* could protect the photosynthesis system from oxidative stress at high temperatures; however, the BR signalling gene *BZR1* could not influence *HSP* expression during heat stress [33,39]. In soybean and rice, *HSFA2* and *HSFA3* enhance heat tolerance by activating the expression of *HSPs* [40,41,42]. WRKY transcription factors have been extensively utilized to enhance heat stress tolerance in crops. AtWRKY25 and AtWRKY39 in *Arabidopsis* influence high-temperature stress tolerance by regulating the transcription levels of *HSFs* and *HSP100* [43,44]. Increased expression of *WRKY11* in rice also resulted in enhanced tolerance to heat [45]. Our study also examined the expression profiles of genes associated with *HSPs*, *HSFs*, and *WRKYs*, and the result was basically consistent with those previous studies. All the analysed genes were expressed equally when plants were grown under normal conditions, and the expression increased in response to heat stratification. P*_SlBRI1_*::S1040A-GFP lines, which were most resistant to high temperature, exhibited the highest accumulation of *HSPs*, *HSFs*, and *WRKYs* (Figure 8). Furthermore, the expression of *SlBRI1* and *SlCPD* showed no obvious regulation during heat stress (data not shown). These data suggested that the modulatory effect of SlBRI1 Ser-1040 on tomato heat tolerance was exerted mainly via changes in the expression levels of *HSPs*, *HSFs*, and *WRKYs*. Notably, in contrast to P*_SlBRI1_*::S1040A-GFP, the heat-responsive modulatory mechanism in *cu3^-abs1^* was mostly associated with BR signal strength, since *cu3^-abs1^* plants with high tolerance to heat stress showed similar expression levels of *HSPs*, *HSFs*, and *WRKYs* as P*_SlBRI1_*::S1040D-GFP and P*_SlBRI1_*::SlBRI1-GFP plants, and the expression of *SlBRI1* and *SlCPD* increased and decreased, respectively, in response to heat stress (Figure 8 and Figure S4).

Tomato is a major horticultural crop that is thermophilic but cannot withstand very high temperatures; therefore, tomato plants cannot survive the high temperatures during the summer in most tomato planting regions, which shortens the growing season. Thus, studies on the mechanism of heat tolerance in tomato to improve yield under high temperature are very important in tomato breeding. Previous studies have demonstrated that both plant growth and fertilization, which are determinants of plant yield, are sensitive to ambient temperature and are usually inhibited when the temperature increases [46]. The phytohormone BR is involved in plant promotion and stress adaption, and BR-treated plants exhibit improved photosynthetic activity, total chlorophyll content, and membrane integrity, which together promote tomato yield under heat stress [47]. The BR receptor BRI1 has been found to play an important role in crop yield via regulation of plant architecture. Several *bri1*-defective mutants of many species exhibit a semi-dwarf phenotype and decreased stem-leaf angles, which are recognized as indispensable attributes for intensive agriculture [15,19,48]. We previously reported that SlBRI1-overexpressing plants exhibited increased tomato yields, and dephosphorylated Thr-1050 of SlBRI1 could promote tomato yield by compacting plant architecture, as well as increasing fruit number and fruit weight, under normal conditions [16,25]. In the present investigation, the effects of Ser-1040 of SlBRI1 on tomato yield were distinct from those of Thr-1050. Ser-1040 had no effects on plant development under normal conditions but could improve tomato yield under heat stress. The early yield of individual P*_SlBRI1_*::S1040A-GFP plants was similar to that of P*_SlBRI1_*::SlBRI1-GFP and P*_SlBRI1_*::S1040D-GFP plants; however, the yield of P*_SlBRI1_*::S1040A-GFP at the late-spring stage with high temperature was significantly higher than that of other lines, mainly due to an increase in number of flowers and in the fruit setting rates (Figure 3). Further investigation indicated that this increase in the fruit setting rate occurred regardless of plant fertilization, since the flower phenotype was unchanged and similar (Figure 3 and Figure S1), and the pollen viability, which is usually sensitive to high-temperature stress. was also the same among all of the transgenic plants. Thus, we concluded that Ser-1040 could increase the yield at high temperatures based on the following aspects. First, the increased yield of P*_SlBRI1_*::S1040A-GFP lines might be related to their excellent photosynthetic capabilities under heat stress, as the decline rates of both the total chlorophyll content and CO_2_ assimilation rates in P*_SlBRI1_*::S1040A-GFP lines were less than those in P*_SlBRI1_*::SlBRI1-GFP and P*_SlBRI1_*::S1040D-GFP lines under heat stress (Figure 7). Second, the increased activity of the ROS detoxification system under heat stress might increase the yield of P*_SlBRI1_*::S1040A-GFP lines. The activities of several enzymes, such as SOD and POD, were higher in P*_SlBRI1_*::S1040A-GFP lines than in other transgenic lines, and these increased activities were caused by an increase in *Cu/Zn-SOD* and *POD1* gene expression levels (Figure 6 and 8). Third, compared with the levels in P*_SlBRI1_*::SlBRI1-GFP and P*_SlBRI1_*::S1040D-GFP lines, the high expression levels of *HSPs*, *HSFs*, and *WRKYs* in P*_SlBRI1_*::S1040A-GFP also helped increase the yield of this line under heat stress (Figure 8). Furthermore, to confirm that the higher yield at later stages in P*_SlBRI1_*::S1040A-GFP lines was indeed due to their tolerance to high temperature rather than their phenotypes at different developmental stages, we delayed the seeding times of all the plants for two months to ensure that their seedling stages occurred entirely under high-temperature conditions. The results showed that P*_SlBRI1_*::S1040A-GFP plants grew stronger and had more flowers in the first two inflorescences than plants of P*_SlBRI1_*::SlBRI1-GFP and P*_SlBRI1_*::S1040D-GFP lines (Figure S3). Taken together, our results clearly demonstrated that SlBRI1 Ser-1040 could influence the capacities of photosynthesis and ROS detoxification, as well as elevate the expression of heat defense-related genes in tomato, all of which functioned together to maintain stable high yields under high-temperature conditions.

## 4. Materials and Methods

### 4.1. Sequence Alignment and Phylogenetic Analysis

The multiple amino acid sequence alignment of BRI1 was performed in ClustalX2 (ftp://ftp.ebi.ac.uk/pub/software/clustalw2/2.1/) with default parameters. The phylogenetic analysis was performed using MEGA7 software (The Pennsylvania State University, State College, Pennsylvania, USA) and the neighbour-joining method and Poisson substitution model with 1000 bootstrap replications. The motif analysis was performed using MEME software (http://meme-suite.org/tools/meme) with the following parameters: (1) the optimum motif width was between 6 wide and 30 wide; (2) the maximum number of motifs was 30; and (3) the other parameters were set to the default values.

### 4.2. Generation of Transgenic Plants by Site-Directed Mutagenesis

Both the native promoter (2989 bp) of *SlBRI1* (Solyc04g051510) and its coding sequence without a stop codon were amplified from tomato (*Solanum lycopersicum* cv. Moneymaker). The amplified fragments were then recombined into the plant expression vector pBI121 (CLONTECH, Palo Alto, CA, USA) with a GFP tag followed by the CT region to construct the vector encoding wild-type *SlBRI1* (P*_SlBRI1_*::SlBRI1-GFP-pBI121). The S1040A or S1040D mutation of SlBRI1 was amplified from P*_SlBRI1_*::SlBRI1-GFP-pBI121 using overlap PCR amplification to obtain the plant expression vector P*_SlBRI1_*::S1040A-GFP-pBI121 or P*_SlBRI1_*::S1040D-GFP-pBI121, respectively. All the constructed vectors were transformed into *Agrobacterium tumefaciens* GV3101 (WEIDI, AC1001S, Shanghai, China) for tomato transformation. Primers designed for plant expression vector construction are listed in Table S1.

For tomato transformation, *cu3^-abs1^* was used in accordance with the cotyledon transformation method [49]. The transgenic plants were screened by kanamycin and PCR analysis, while quantitative real-time PCR and western blot analysis were used to confirm that the transgenic proteins were stably expressed in these plants. The T_2_ generation of two independent homozygous lines from P*_SlBRI1_*::S1040A-GFP-pBI121 (S1040A-8 and S1040A-10), P*_SlBRI1_*::S1040D-GFP-pBI121 (S1040D-1 and S1040D-2), and P*_SlBRI1_*::SlBRI1-GFP-pBI121 (SlBRI1-1 and SlBRI1-3) were used in this study.

### 4.3. Agronomic Trait Characterization

For agronomic trait investigations, both transgenic lines and *cu3^-abs1^* were planted in early spring and grown in a glasshouse without additional temperature and light controlling equipment. The plant height was measured as the distance from the cotyledon to the top of the plant, while the stem diameter was measured as the diameter of the cotyledon node at the flowering stage of the fourth inflorescence. Single-fruit weight was considered as the individual fruit weight at the RR stage. The early yield was the total weight of the first to the third fruit nodes per plant, while the total yield was the total weight of the first to the sixth fruit nodes per plant. Each agronomic trait was measured for at least 10 independent biological replicates.

### 4.4. Growth Response of Hypocotyls to Exogenous BRZ and BL

To analyse the growth response of hypocotyls to BR signalling, sterilized seeds of both transgenic lines and *cu3^-abs1^* were inoculated in Petri dishes that contained solid 1/2 strength Murashige and Skoog medium (1/2 MS medium) with BRZ (Tokyo Chemical Industry Co., Ltd., Tokyo, Japan) at 0 nM, 10 nM, 100 nM, 500 nM, and 1000 nM or with epi-BL (Shanghai Yuanye Biotechnology Co. Ltd., Shanghai, China) at 0 nM, 10 nM, 100 nM, 500 nM, and 1000 nM. These seeds were maintained at 25 °C in the dark for 10 days. Hypocotyl lengths were subsequently measured, and the relative hypocotyl length represented the changes in the hypocotyl length at different concentrations of BRZ or epi-BL. Each treatment was performed on at least 15 seedlings.

### 4.5. Germination Analysis of Plants under Heat Stress

For germination assays under heat stress, the seeds of both transgenic lines and *cu3^-abs1^* were inoculated in Petri dishes with wet filter paper under 33 °C or 28 °C (control), a 16 h light/8 h dark cycle, and 250 μmol⋅m^−2^⋅s^−1^ photosynthetic photon flux density (PPFD) (BN058C LED11/WW L1200 GC OL, Philips, Amsterdam, Holland) as previously reported [50]. The germination rate and germination potential were calculated on the fourteenth day and fourth day, respectively. The root length and MDA content of early-stage seedlings were both detected on the fourteenth day after seeding. There were three replicates for each index, and each replicate had 30 plants.

### 4.6. Heat Treatment of Seedlings

To analyse the seedling tolerance to heat stress, tomato seeds of both transgenic lines and *cu3^-abs1^* were sown in plastic pots (15 × 15 × 15 cm) containing peat (PH 6.0, Pindstrup Plus, Pindstrup Rosebrug A/S Denmark) and vermiculite (8:2, v/v) under the following conditions: 16 h light/8 h dark photoperiod, 25 °C, 70% relative humidity (RH), and 250 μmol⋅m^−2^⋅s^−1^ PPFD. For heat shock stress, seedlings at the four-leaf stage were exposed to 38 °C/28 °C (day/night) for 9 days and then transferred to 25 °C for 3 days, with other environmental conditions remaining unchanged. The control seedlings were maintained at 25 °C during the same period. Each index was measured for at least 3 independent biological replicates.

### 4.7. Growth Performances of Seedlings under Heat Stress

Both the fresh weight and dry weight of the shoots and roots, as well as the seedling height and seedling stem diameter, were measured on the ninth day during heat stress. For seedling dry weight measurement, the seedlings were dried at 105 °C for 15 min and then placed at 75 °C to achieve constant weight. There were 3 replicates, and each replicate had 10 plants.

### 4.8. Physiological Parameter Measurements

The third fully expanded leaves from the top of the control and stressed seedlings were harvested for physiological parameter measurements at 0, 3, 6, 9, and 12 days during heat stress. The MDA content, electrolyte leakage, and free proline accumulation were determined in accordance with the methods described by Liu [51]. The chlorophyll content and CAT, SOD, and POD activities in leaves were measured as described previously [17]. The CO_2_ assimilation rate of third fully expanded leaves from the top was measured by using an infrared gas analyser-based portable photosynthesis system (LI-6800; LI-COR, Lincoln, NE, USA), the measurements were carried out at 25°C, 800 μmol⋅m^−2^⋅s^−1^ light intensity, 70% RH, and 400 μmol mol^-1^ CO_2_ concentration, respectively. The chlorophyll content were measured according to the methods described by Kong [52]. Each index had at least 3 replicates, and each replicate had 10 plants.

### 4.9. DAB Staining

The upper-third fully expanded leaves of the control and stressed seedlings were stained by a solution containing 1 mg ml^–1^ 3,3′-diaminobenzidine (DAB, D12384, Sigma-Aldrich, Saint Louis, MO, USA), pH 5.5 to detect the accumulation of H_2_O_2_ in leaves. The histochemical analysis of H_2_O_2_ was performed as described previously [33].

### 4.10. Autophosphorylation Analysis

For autophosphorylation analyses in vitro, the cytoplasmic domain (824 to 1207 aa) of SlBRI1, S1040A, S1040D, or K916E was amplified from P*_SlBRI1_*::SlBRI1-GFP-pBI121, P*_SlBRI1_*::S1040A-GFP-pBI121, P*_SlBRI1_*::S1040D-GFP-pBI121, or P*_SlBRI1_*::SlBRI1-GFP-pBI121 with the specific primers (*SlBRI1*-CD-F and *SlBRI1*-CD-R) listed in Table S1. The amplified fragment was subcloned into the prokaryotic expression vector pFLAG-MAC and then transformed into *E. coli* BL21 (DE3) pLysS (Transgene, CD901-02, Beijing, China). SlBRI1-FLAG-MAC and K916E-FLAG-MAC were used as the positive and negative controls, respectively, and subsequent protein purification and autophosphorylation analyses in vitro were performed according to previously described methods [21,53]. Intensities of bands were quantified by using ImageJ software (NIH, Bethesda, Maryland, USA) and presented as relative values compared with the FLAG-SlBRI1.

### 4.11. Western Blot Analysis

Target proteins were extracted from 4-week-old transgenic tomato leaves (0.2 g) expressing SlBRI1, S1040A, or S1040D, and the experiment was performed as previously described [21].

### 4.12. Quantitative Real-time PCR Analysis

The second leaves from the top of the seedlings were selected for transcription analysis at 0, 3, 6, 9, and 12 days during heat stress. Total RNA from the leaves of the seedlings was extracted with an RNAiso Plus Kit (TaKaRa, 9109, Kusatsu, Japan) and transcribed to cDNA with a Transcriptor First Strand cDNA Synthesis Kit (Roche, 4896866001, Mannheim, Germany). qRT-PCR was performed by using a SYBR Green Master Mix Kit (Vazyme, Q121-02, Nanjing, China). The method was performed according to the manufacturer’s protocol, and primers for target genes were designed based on previously described methods [33,54]. The tomato housekeeping gene *Actin* was amplified as an internal reference. Each data point had 3 biological replicates and technical replications.

### 4.13. Statistical Analysis

The data in this study were analysed using a one-way analysis of variance (ANOVA) via SPSS 17.0. (IBM, Armonk, New York, USA). The means and standard errors of each sample were calculated, significant differences between means were examined by LSD test (*p* < 0.05) and represented by different letters.

## 5. Conclusions

Heat stress is a major abiotic stress influencing agricultural production, and BRI1 is a BR receptor that plays a critical role in plant growth and stress adaption. Previous studies of SlBRI1 phosphorylation sites have suggested that modifying SlBRI1 Thr-1050 in tomato could promote yield through precise control of BR signal strength; however, this functional analysis was limited to Thr-1050, and the associated agronomic traits of other phosphorylation sites, especially their functions in stress tolerance, remained unclear. In this study, we revealed for the first time the biological role of SlBRI1 phosphorylation sites in tomato stress adaption. SlBRI1 Ser-1040 was found to participate in plant responses to heat stress by influencing the autophosphorylation of SlBRI1. Transgenic plants harbouring S1040A exhibited better performance, including faster growth rates, better germination, more active photosynthetic and ROS detoxification systems, and higher expression of heat defence genes, under high temperature, which helped them adapt to and maintain high yields under heat stress. Our results not only shed light on the molecular mechanisms of SlBRI1 in stress adaption regulation but also provide a molecular basis for establishing high-yield tomato lines via fine-tuning of phosphorylation sites of SlBRI1.

## Supplementary Materials

Supplementary Materials can be found at https://www.mdpi.com/1422-0067/21/20/7681/s1. Figure S1: Yield trait analysis of *cu3^-abs1^* and transgenic plants harbouring SlBRI1, SlBRI1 dephosphorylated at Ser-1040, and SlBRI1 phosphorylated at Ser-1040; Figure S2: Dephosphorylation of Ser-1040 promotes seedling growth under heat stress; Figure S3: Dephosphorylation of Ser-1040 improves tomato growth and flower number under heat stress; Figure S4: BR signalling influences the heat tolerance of *cu3^-abs1^*; Table S1: Primers used in this research.

## Abbreviations

BR	Brassinosteroid
BRI1	Brassinosteroid Insensitive1
CPD	Constitutive Photomorphogenesis and Dwarf
epi-BL	24-Epibrassinolide
BRZ	Brassinazole
K916E	Lysine-916-Glutamic acid
GFP	Green fluorescent protein
Ser	Serine
S1040A	Serine-1040-alanine
S1040D	Serine-1040-aspartic acid
Thr	Threonine
Tyr	Tyrosine
ROS	Reactive oxygen species
DAB	3,3′-Diaminobenzidine
CAT	Catalase
SOD	Superoxide dismutase
RBOH1	RESPIRATORY BURST OXIDASE HOMOLOG1
HSF	Heat shock transcription factor
HSP	Heat shock protein
POD	Peroxidase
MDA	Malondialdehyde
Pn	Net photosynthetic rate
qRT-PCR	Quantitative real-time PCR analysis
MS	Murashige and Skoog medium
PPFD	Photosynthetic photon flux density
