# Supplementary material for: Modification of Serine 1040 of SIBRI1 Increases Fruit Yield by Enhancing Tolerance to Heat Stress in Tomato
Source: Int J Mol Sci. 2020 Oct 16;21(20):7681. doi: 10.3390/ijms21207681 (PMC7589314; doi:10.3390/ijms21207681)
Supplement: Supplementary file 1 [file ijms-21-07681-s001.pdf]

International Journal of
Molecular Sciences

MDPI

Supplementary figures

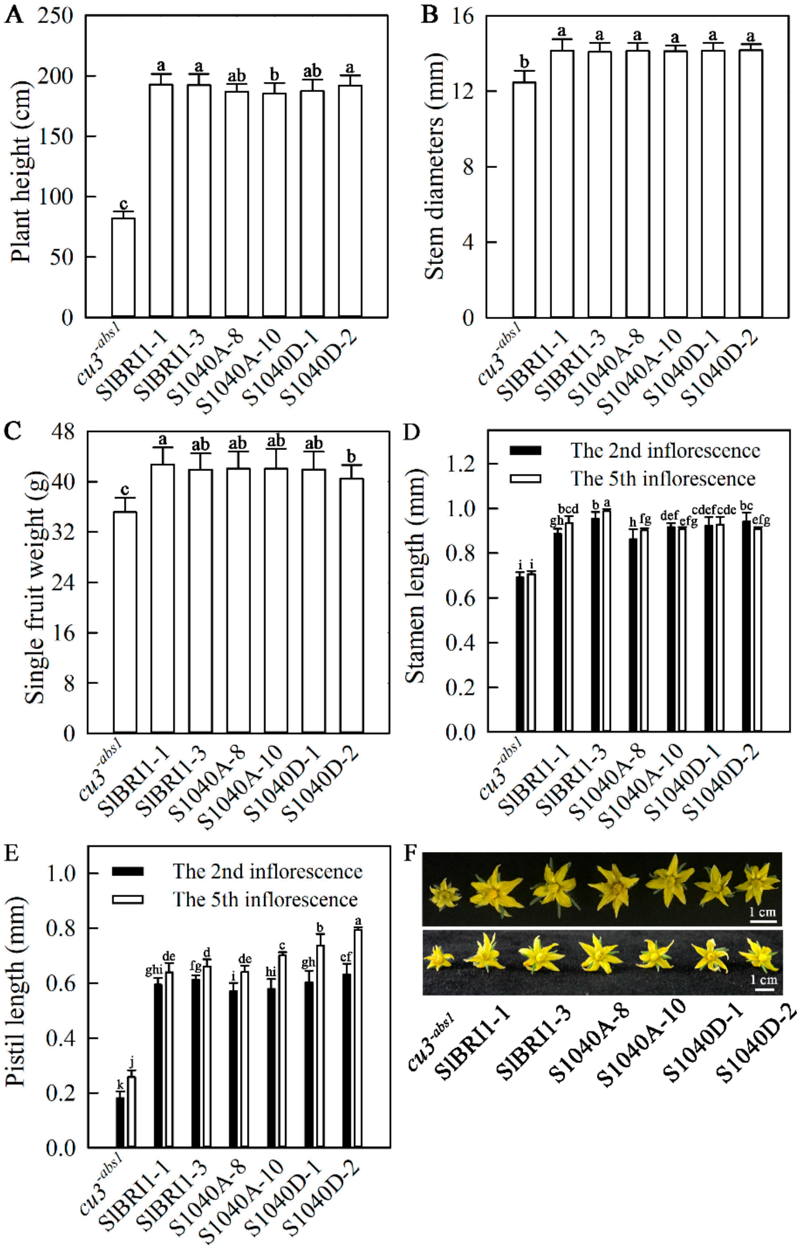

Figure S1. Yield trait analysis of *cu3*$^{-abs1}$ and transgenic plants harbouring SlBRI1, SlBRI1 dephosphorylated at Ser-1040, and SlBRI1 phosphorylated at Ser-1040. (A) Plant height. (B) Stem diameter. (C) Single-fruit weight. (D-F) Stamen length (D), pistil length (E), and flower phenotype (F) of the second and fifth inflorescences. The data for (A-E) are the means ± SDs of at least 10 independent biological samples. The different letters in (A-C) and (D-E) indicate significant differences compared with P_{SlBRI1}::SlBRI1-GFP-1 plants and the second inflorescence of P_{SlBRI1}::SlBRI1-GFP-1 plants, respectively ($P < 0.05$).

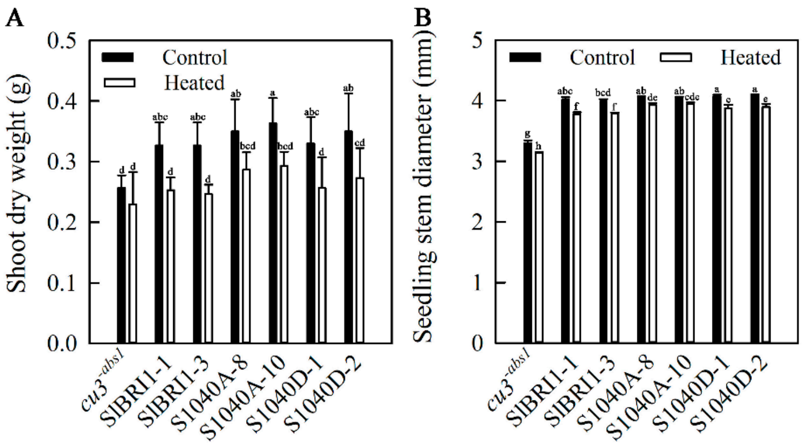

Figure S2. Dephosphorylation of Ser-1040 promotes seedling growth under heat stress. (A) Shoot dry weights and (B) seedling stem diameters of plants at the four-leaf stage treated with or without heat stress (38°C/28°C, day/night) for 12 days. The data are the means ± SDs of 3 independent biological samples. The different letters indicate significant differences compared with P_{SlBRI1}::SlBRI1-GFP-1 plants grown under normal conditions (P< 0.05).

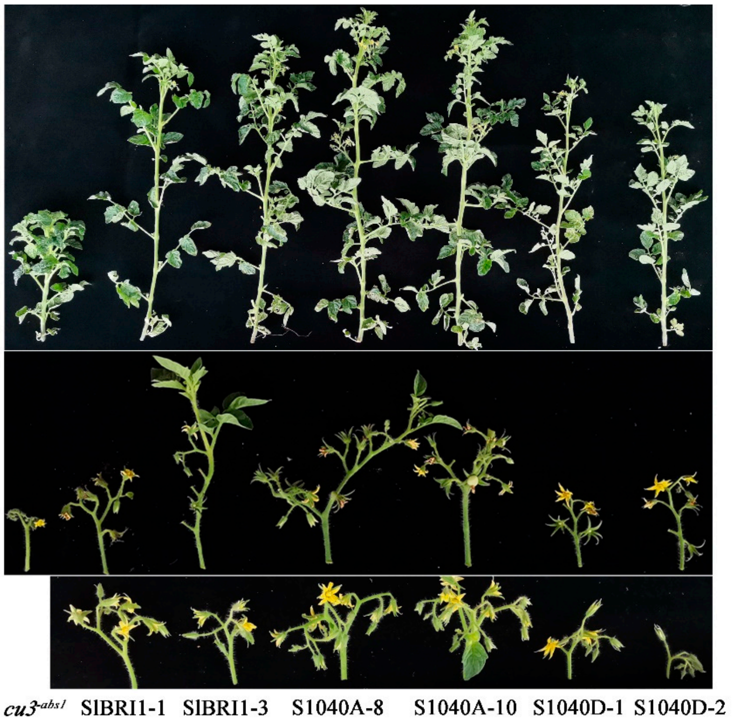

Figure S3. Dephosphorylation of Ser-1040 improves tomato growth and flower number under heat stress. Phenotype of the plant (top), first inflorescence (middle), and second inflorescence (bottom). Plant seeding was delayed by two months at the late-spring stage.

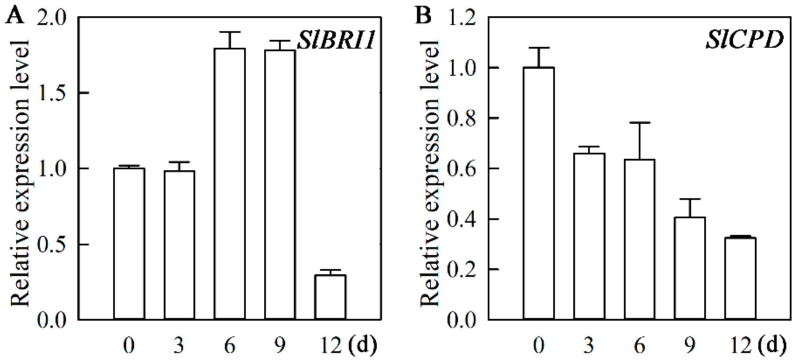

Figure S4. BR signalling influences the heat tolerance of *cu3*$^{-abs1}$. (A) and (B) Relative transcript levels of *SlBRI1* (A) and *SlCPD* (B) in *cu3*$^{-abs1}$ plants under heat stress. The *cu3*$^{-abs1}$ seedlings at the four-leaf stage were placed into a growth chamber set at 38°C/28°C (day/night) for 9 days and then transferred to 25°C for 3 days. The data are the means ± SDs of 3 biological replicates and technical replications.

Table S1. Primers used in this research

Gene	Forward primer (5′-3′)	Reverse primer (5′-3′)
Promoter of SlBRI1	GACCATGATTACGCCAAGCTTCTCC ATTTCAATTATTGCTCAAAGG	ACCACCCGGGGATCCTCTAGACTTC AAAGATTGAAACTTTATAGCTTAAA
SlBRI1 for tomato transformation	TCTTTGAAGTCTAGAGCTCGAGATG AAAGCTCACAAAACTGTGTTTAAC	GCCCTTGCTCACCATGGTACCCTTAT CGTCGTCATCCTTGTAATC
S1040A for site mutation	GCAAGGTTAATGGCTGCTATGGACA	TGTCCATAGCAGCCATTAACCTTGC
S1040D for site mutation	GCAAGGTTAATGGATGCTATGGACA	TGTCCATAGCATCCATTAACCTTGC
K916E for site mutation	GAGTGTTGTAGCTATTGAGAAATTG ATACACG	CGTGTATCAATTTCTCAATAGCTACA ACACTC
SlBRI1 and slbri1 for phosphorylation analysis in vitro	GACGATGACAAAGTCAAGCTTGAGA CGAAGAAGAGGAGGAGG	TCTGCAGGTACCCGGGAATTCAAGG TGTTTGCTCAGCTCATTG
SlBRI1 for Q-PCR	TTCAATGGCACGATCCCGAA	TGGGGAGAGGATACCCACAG
SlCPD for Q-PCR	ATCCAATTAACGTCCAACAT	ACCTTTCATACACCTCCCTC
RBOH1 for Q-PCR	CTCCAGCACAAGATTACCGA	TCCTCCATTGTGACGATGTT
CAT1 for Q-PCR	TGATCGCGAGAAGATACCTG	CTTCCACGTTCATGGACAAC
Cu/Zn-SOD for Q-PCR	GGCCAATCTTTGACCCTTTA	AGTCCAGGAGCAAGTCCAGT
POD1 for Q-PCR	GTCCGGGAGTTGTTTCTTGT	ATCACCATTGGCTTCTGACA
HSFA2 for Q-PCR	CAATGTCAGGCCGGATTCTG	CTACTTCCTCTGCTGCTCGA
HSFA3 for Q-PCR	CAGACTTTGGTGTTAGCTCTGG	CAGCTGGCCACTTATCGAC
HSP70 for Q-PCR	GGAGAAGAGAGAAGCCATAGA	TCAGCAGGCACCTTATCT
HSP90 for Q-PCR	ATCTTGTGCTGCTGCTGTTTG	GCTTCCTCTTCTTCGTCAATGC
WRKY1 for Q-PCR	CTAGTGCAGGGTCAAGGAAA	ACAGGACTCTTCGTCACTCG
WRKY72 for Q-PCR	AAGCAGGTTCAAAGATGTGC	CAGTTACTTGTGGGTTTGGG
ACTIN for Q-PCR	GTGTGGGCTCACCTACGTTT	ACAATCCCAAGGGTTGTCAC
